# Fetus in Fetu: A Case of Vanishing Triplet Phenomena

**DOI:** 10.7759/cureus.30342

**Published:** 2022-10-16

**Authors:** Bayan Hasan, Mohamed Ebrahim

**Affiliations:** 1 General Practice, KIMSHEALTH Hospital, Manama, BHR; 2 Radiology, Salmaniya Medical Complex, Manama, BHR

**Keywords:** magnetic resonance imaging, explorative laparotomy, intra-abdominal, multiplanar reformation, computed tomography (ct ), breech presentation, twin neonate, fetus-in-fetu, abdominal radiology, general radiology

## Abstract

Fetus in fetu (FIF) is a rare congenital disorder in which a deformed fetus forms inside a normal one, mostly within the abdominal cavity. In this case study, we present a triplet pregnancy where one fetus was formed within a viable fetus. This was noted in prenatal imaging and upon delivery. Radiological investigations were conducted, including plain X-rays, abdominal computed tomography, and magnetic resonance imaging, and a preliminary case of FIF was diagnosed. Following this, surgical resection was planned and done soon after birth with histopathology confirmation of diagnosis, and it revealed no evidence of somatic malignancy. Currently, the patient is in stable condition and is being followed up with serial ultrasound imaging with alpha-fetoprotein levels to detect recurrence.

## Introduction

Fetus in fetu (FIF) is a rare congenital malformation with less than 200 cases reported worldwide and an incidence of one per 500,000 births [1]. It involves a diamniotic monochorionic fetal twin forming inside the body of its own normally developed twin, most commonly within the abdominal cavity [1]. The first mention of this unusual condition in literature was discovered by Meckel in the late 18th century [1].

This condition has been described in both pediatric and adult populations; however, it appears more commonly during early childhood with a 2:1 male-to-female ratio [2]. Furthermore, both single and multiple fetuses within a single fetus have been previously reported, in which the mother carries a single living fetus encasing the other smaller demised fetus [2]. Our case is unique because this condition appeared as a part of a triplet pregnancy, which resulted in two viable babies and the third baby was found encased in the body of the largest living newborn.

In this report, we present a rare case of FIF in a four-month-old female infant, who was a product of an in vitro fertilization (IVF) twin pregnancy to a primigravida mother. To the best of our knowledge, this is the first case of FIF to be reported within our institution as well as in our country of practice, Bahrain.

## Case presentation

In this case, we report a four-month-old female infant who was the product of an elective lower segment C-section (LSCS) due to twin pregnancy with a breech presentation of the first twin. The pregnancy was conceived through IVF by a 30-year-old primigravida woman. During her pregnancy, an antenatal abdominal ultrasound demonstrated an abdominal cystic lesion with ascites in one fetus.

**Figure 1:**
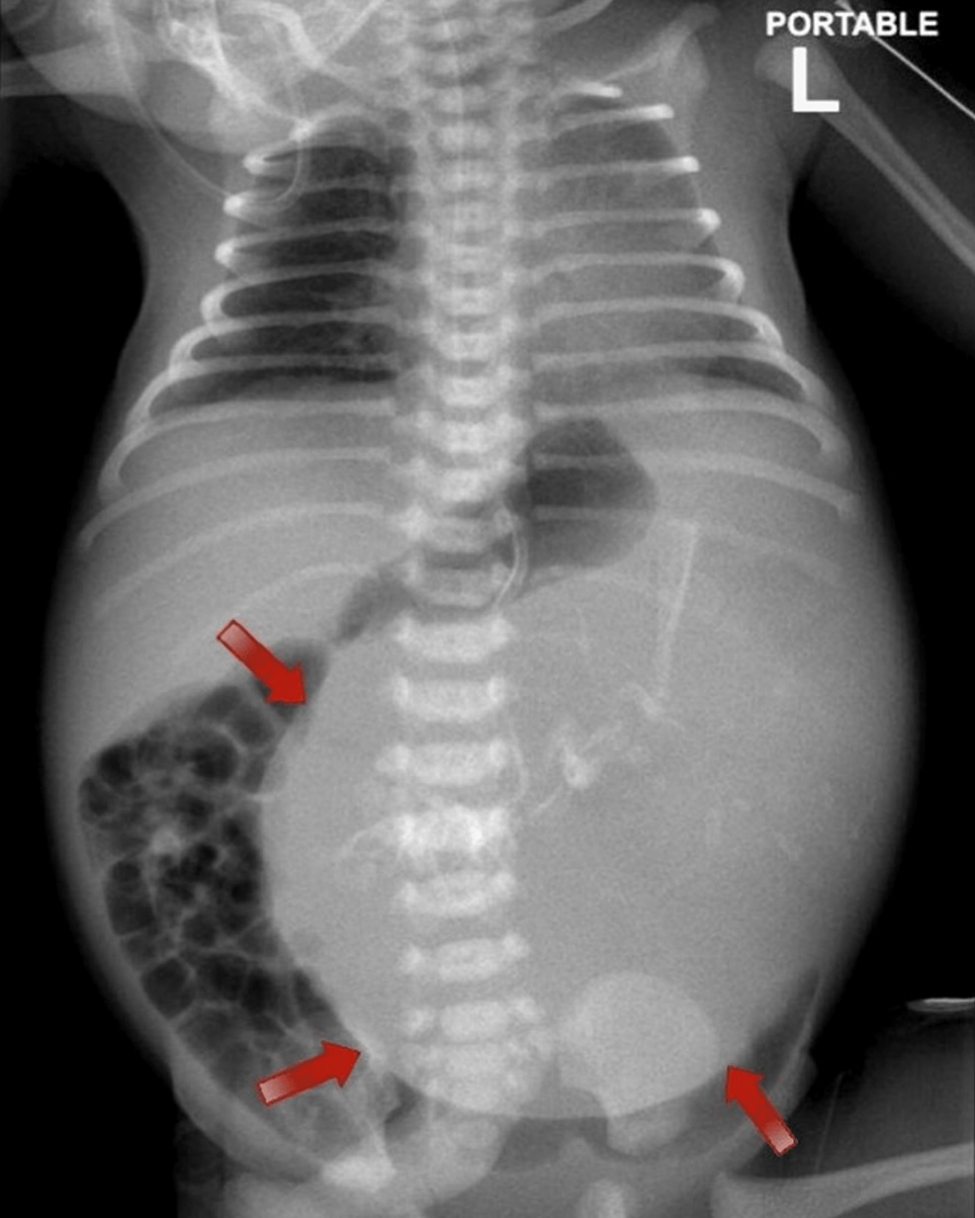
Anteroposterior abdominal X-ray demonstrating the abdominal mass crossing the midline and displacing the bowel

Our twin of interest, in this case, had an APGAR (appearance, pulse, grimace, activity, and respiration) score of 9, 10, and 10 upon birth. Noticeably, following her first postnatal examination, she appeared normal without any dysmorphic features. However, her abdomen appeared to be distended on inspection. Further focused physical examination showed a large abdominal mass in the left hypochondrium crossing the midline. Following this, she was admitted into the neonatal intensive care unit (NICU) for further investigations. The neonate was kept nil per oral (NPO) and kept on fluid maintenance until the workup was completed. Initially, plain abdominal radiographs demonstrated a large cystic shadow in the left hypochondrium with radio-opaque shadows, as seen in Figure 1.

**Figure 2:**
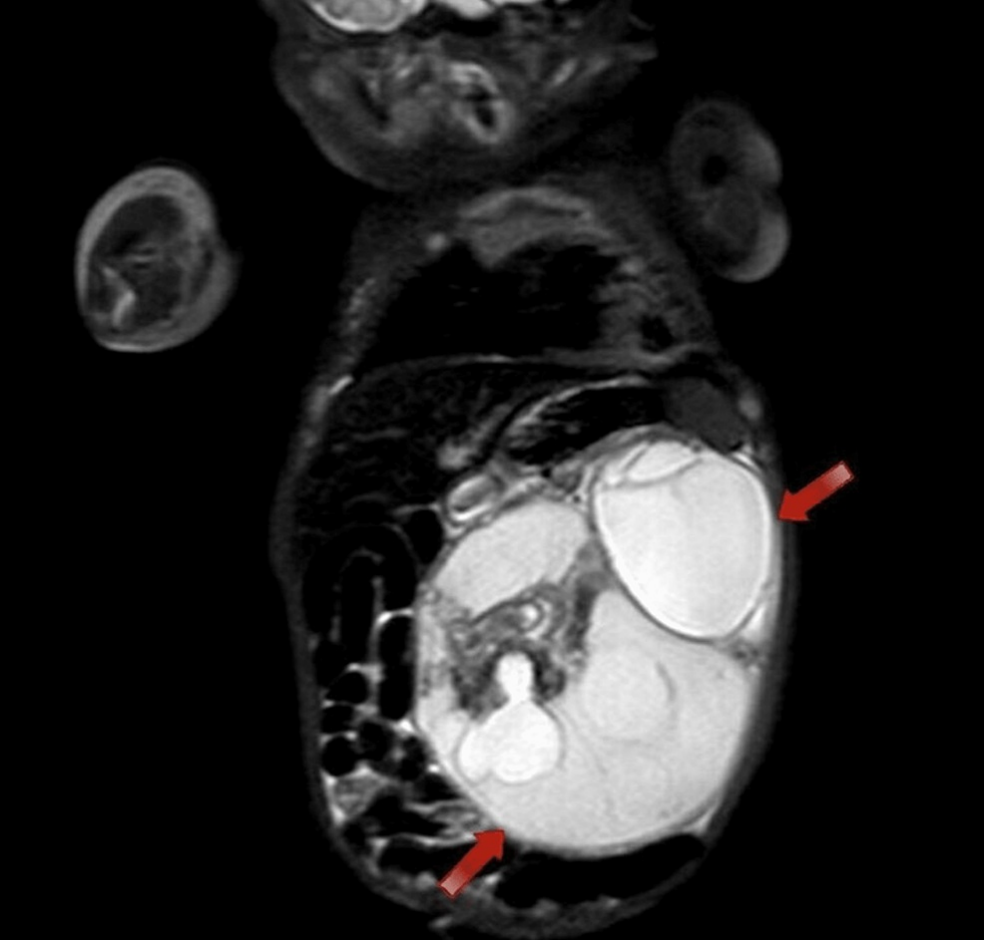
MRI coronal plane view showing the large mass with soft tissue and cystic components

**Figure 3:**
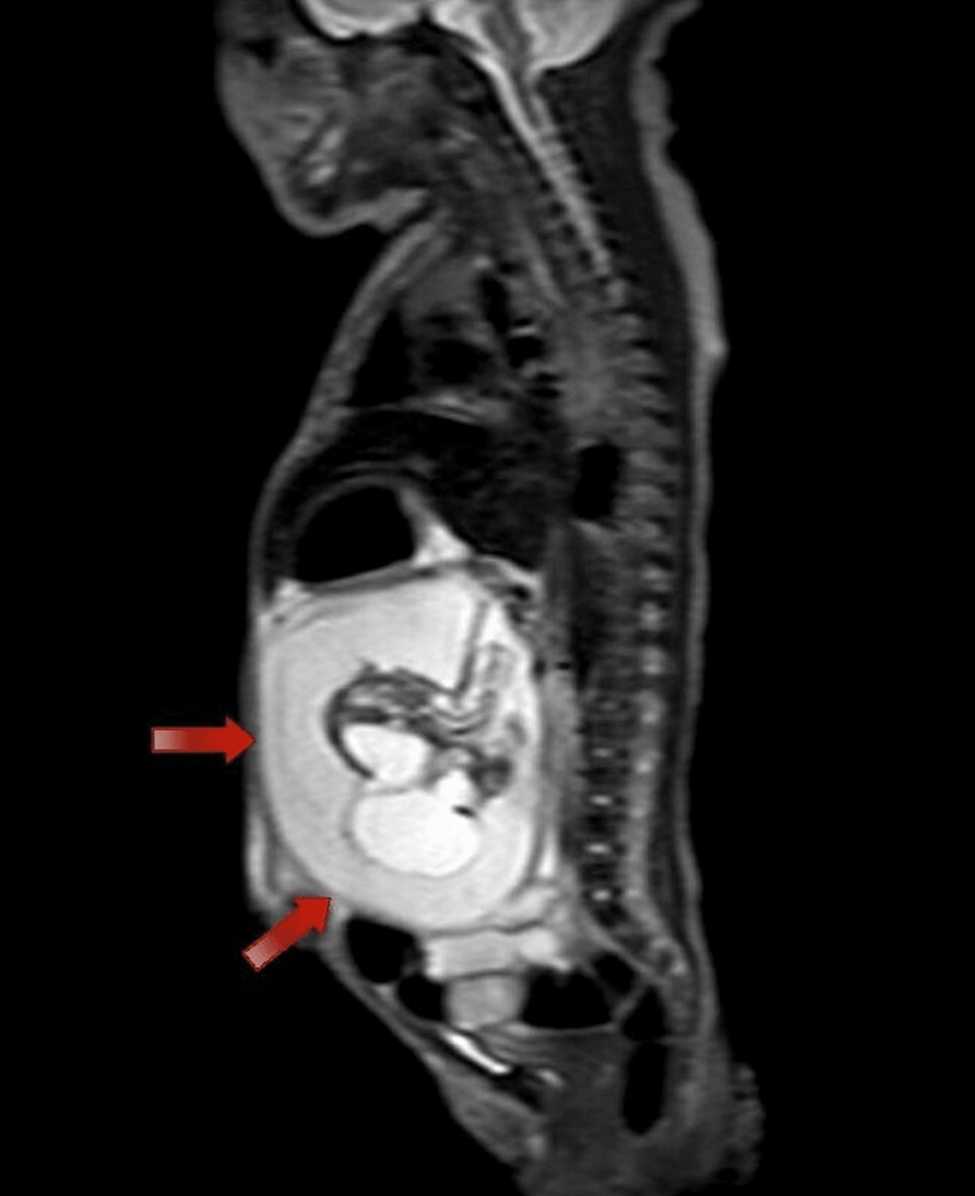
CT sagittal plane demonstrating the fetus with soft tissue and bony components

Moreover, a computed tomography (CT) scan of the abdomen and pelvis and a magnetic resonance imaging (MRI) scan were done, which showed a large complex mass occupying most of the peritoneal cavity, mainly located on the left side. Images showed that the mass contained fat, fluid, soft tissues, and parts of the fetal skeleton and limbs, along with neural tissues, with the mass having an arterial supply from the aorta. These findings can be seen in Figures 2, 3.

**Figure 4:**
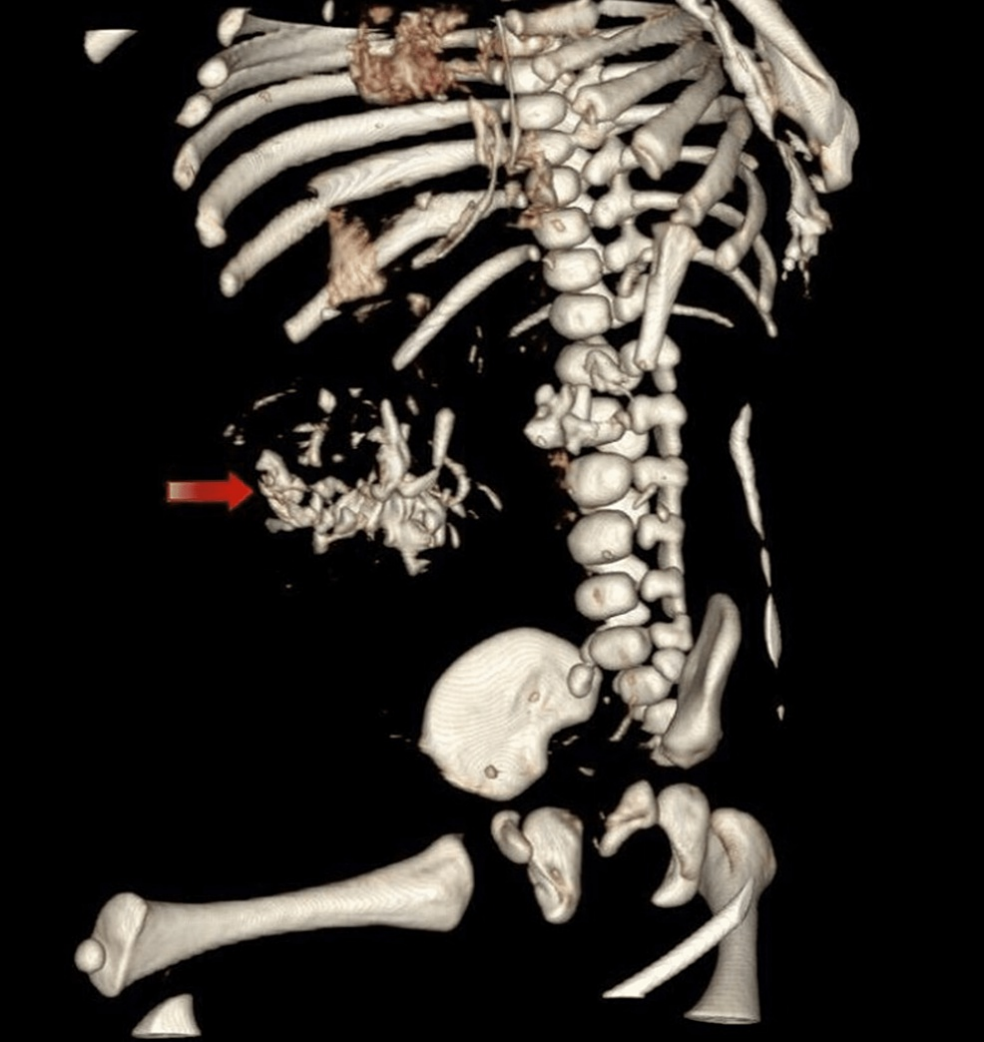
Three-dimensional skeletal reconstruction showing fetal bones within the infant's abdominal cavity

Furthermore, a three-dimensional (3D) reconstruction of the bony skeleton was done, demonstrating the fetal skeletal bones and limbs present within the abdominal cavity of the infant, as seen in Figure 4.

Consequently, the surgical team performed a laparotomy on the infant to resect the mass along with its capsule. Upon gross examination following resection, the mass had a well-defined capsule and multiple large vessels feeding it, which corresponded to the imaging reports. It weighed 366 grams, and its contents were bone, hair, and fetal limb remnants, all of which were further taken for histopathology. The histopathology report showed findings consistent with FIF malformation with no evidence of somatic malignancy.

The patient’s postoperative period was uneventful, in which she was extubated and discharged in good condition. Concurrently, preoperative tumor markers were sent, but the sample was insufficient, and no readings were available. One week after the operation, alpha-fetoprotein (α-FP) was repeated, and the result came elevated, measuring 36964.4 micrograms/L. However, follow-up α-FP levels showed regression, with the latest level measuring around 14136.5 micrograms/L at six months postoperatively. A follow-up abdominal ultrasound was done one-year postoperatively and showed no significant abnormal soft tissue at the site of the surgery.

## Discussion

When discussing FIF, multiple theories arise regarding the pathogenesis of this rare condition. One theory is the uneven division of the developing blastocysts’ totipotent cells, resulting in the encasement of the smaller product of the division into the larger maturing embryo [3]. This leads to the encasement of the diamniotic monochorionic twin within the larger viable twin [3]. This theory could explain the findings in our patient. Another theory suggests that it is a benign growth that is primarily considered a progression of a teratoma [4] However, it is important to distinguish FIF from teratomas since the latter condition has a distinct malignant potential in contrast to FIF, which is benign in origin [4]. One distinguishing feature that helps radiologists distinguish FIF from a teratoma is the appearance of a vertebral column in the former condition, with only one case in the literature reported by Hopkin et al., in which a patient with FIF developed malignant transformation [4].

Typically, FIF is an intra-abdominal pathology, although some cases have reported this pathology being located within the cranial cavity or even the scrotum [5]. The initial radiological investigation for patients who present with an abdominal mass is typically a plain abdominal radiograph. In cases of FIF, a plain X-ray can also aid in diagnosis if the spinal axis within the mass is apparent. Another ancillary feature is the lesion’s location, where a teratoma is mainly seen in the lower pelvis, ovaries, and sacrococcygeal regions. Alternatively, FIF is usually seen in the upper retroperitoneal region [5,6]. In addition, a prenatal abdominal ultrasound can aid in the diagnosis, as seen in a study by Nicolini et al., who first wrote about a FIF found on prenatal ultrasound in 1983 [7].

Additionally, a contrast-enhanced CT scan can help to further characterize the mass and can be of great value in assessing the surrounding structures and vascularity for planning the surgical resection. The typical CT findings of FIF include a rounded or tubular mass with a fat component surrounding the vertebral bony axis [8]. Moreover, intra-abdominal FIF is usually seen encased by a separate sac without vascular connections to the host, although several cases reported vascular supply in the form of vascular plexus arising from the attached abdominal wall [8].

MRI is currently being used more frequently to diagnose this entity due to its ability to provide detailed tissue characterization. Further, its lack of ionizing radiation makes MRI a safe tool to confirm the diagnosis in the antenatal phase and during follow-up in the postnatal period. It can provide a thorough evaluation of the bony structures, including the vertebral bodies, rib-like and long bones, limb buds with primitive phalanges, and the cranial fossa and orbital sockets.

Although not as common as a CT or MRI scan, reconstructive tools such as multiplanar reformation (MPR) and volume rendering technique (VRT) can provide 3D images and aid in the evaluation of the surrounding anatomical structures and the exact location of the lesion, as was done in our case. FIF’s diagnosis primarily depends on radiological imaging and a definitive diagnosis can be made by histopathology following resection, markers such as α-FP levels can also help in this condition’s diagnosis and follow-up [8]. Despite the possibility that α-FP may be normal during the early stages of the condition, it does not preclude significant pathology. It can also be used to detect a recurrence on follow-up after resection [8,9].

The treatment of surgical resection of the encapsulated fetus relieves the obstruction and prevents further compression. Complete surgical resection of the surrounding sac is important since leaving behind a portion of the mass capsule may facilitate recurrence, although a case by Magnus et al. in 1999 reported that it recurred as a yolk sac tumor four months after the operation [9].

When comparing the management of this case with that of other FIF cases worldwide, we notice that the approach is similar to the one undertaken in this case. Most authors report that initial suspicion of the diagnosis begins with antenatal ultrasound within the final trimester of the pregnancy and Mills et al. described the importance of antenatal ultrasound in the diagnosis of FIF [6-9]. This is similar to our case in which the pathology was first noticed using an antenatal ultrasound.

Further investigations usually began with plain radiographs followed by further imaging in the form of a CT scan or an MRI depending on the location of the FIF. Once the preliminary diagnosis was done, complete surgical resection was undertaken during infancy. This was seen in the cases reported by Magnus et al. and Nanjaraj et al., in which the approach was quite identical to that of our case even though the locations of the FIF were intrahepatic and intracranial, respectively [5,9].

Finally, frequent postoperative surveillance is recommended, especially if the sac could not be completely resected. This may be achieved with moderate sensitivity using serum tumor marker concentrations and imaging, preferably modalities without ionizing radiation, such as ultrasound or MRI.

## Conclusions

FIF is a rare congenital condition that should be a part of the differential of infants presenting with pelvic-abdominal mass. It has similar imaging and histological features as teratoma; however, the presence of axial and long bones within the mass in radiological imaging is diagnostic of the entity. The ideal imaging modalities to diagnose and plan for surgical intervention are either a CT scan or an MRI. However, given the risks of radiation exposure, MRI is more frequently used both pre- and postnatally.
